# Aiming for quality: a global compass for national learning systems

**DOI:** 10.1186/s12961-021-00746-6

**Published:** 2021-07-19

**Authors:** Diana Sarakbi, Nana Mensah-Abrampah, Melissa Kleine-Bingham, Shams B. Syed

**Affiliations:** 1 Health Quality Programs, Queen’s University, Kingston, Canada; 2 Integrated Health Services, World Health Organization, Geneva, Switzerland; 3 Health Quality Programs, Queen’s University, Cataraqui Building, 92 Barrie Street, Kingston, ON K7L 3N6 Canada

**Keywords:** Learning health system, Health policy, Implementation science, Quality improvement

## Abstract

**Introduction:**

Transforming a health system into a learning one is increasingly recognized as necessary to support the implementation of a national strategic direction on quality with a focus on frontline experience. The approach to a learning system that bridges the gap between practice and policy requires active exploration.

**Methods:**

This scoping review adapted the methodological framework for scoping studies from Arksey and O’Malley. The central research question focused on common themes for learning to improve the quality of health services at all levels of the national health system, from government policy to point-of-care delivery.

**Results:**

A total of 3507 records were screened, resulting in 101 articles on strategic learning across the health system: health professional level (19%), health organizational level (15%), subnational/national level (26%), multiple levels (35%), and global level (6%). Thirty-five of these articles focused on learning systems at multiple levels of the health system. A national learning system requires attention at the organizational, subnational, and national levels guided by the needs of patients, families, and the community. The compass of the national learning system is centred on four cross-cutting themes across the health system: alignment of priorities, systemwide collaboration, transparency and accountability, and knowledge sharing of real-world evidence generated at the point of care.

**Conclusion:**

This paper proposes an approach for building a national learning system to improve the quality of health services. Future research is needed to validate the application of these guiding principles and make improvements based on the findings.

**Supplementary Information:**

The online version contains supplementary material available at 10.1186/s12961-021-00746-6.

## Introduction

Universal health coverage (UHC) is a global health priority that is part of the 2030 Agenda for Sustainable Development endorsed by the United Nations [1]. The goal of UHC is not only to provide access to a national, publicly funded health system but also to deliver quality health services that are effective, safe, people-centred, timely, equitable, integrated, and efficient [2]. There is no single pathway to achieving quality UHC. Each country will need to learn how to transform its health system into a learning one to deliver quality health services to patients, families, and the broader community [3–5]. A learning system is defined by the Institute of Medicine, the most common reference cited in the literature, as a system that learns from itself, where “science, informatics, incentives, and culture are aligned for continuous improvement and innovation, with best practices seamlessly embedded in the delivery process and new knowledge captured as an integral by-product of the delivery experience” [6, 7]. Real-world evidence generated at the point of care could help national health authorities understand the systemic barriers for delivering quality health services and the strategies and policies needed to address them [8, 9].

The WHO Global Learning Laboratory (GLL) for Quality UHC welcomes people from across the world to share their experiences and expertise, challenge each other, and spark innovation for improving the delivery of quality health services. One of the priority areas for the GLL is to share how countries are learning to develop, implement, and refine their national policies and strategies for quality based on the frontline experiences of health professionals and the patients, families, and communities they serve. This strategic approach to learning and decision-making based on context is a continuous cycle of collaboration, feedback, and improvement that requires the engagement of multiple stakeholders across the health system to bridge the gap between practice and policy [10–12].

According to WHO, a country’s health system is formed by the people and organizations who are receiving, delivering, and overseeing health services, consisting of three main levels of stakeholders: (1) patients/families, health professionals, and health organizations, (2) subnational health authorities guiding the delivery of quality health services, and (3) national health authorities setting policies/strategies for quality [13, 14]. A national learning system for improving the delivery of health services involves stakeholders at each level of the health system to form a common vision for quality (organizational, subnational, and national). Having a health system perspective of quality means recognizing that health professionals are not working in silos but are part of a health organization within a subnational and/or national health system that is accountable to patients, families, and the broader community. A national learning system targeting stakeholders across the health system could potentially be effective at implementing sustainable policy changes by uncovering systemic barriers to quality at the point of care [15, 16].

A scoping review was conducted to identify guiding principles for building a national learning system to improve the quality of health services. The first objective of the scoping review was to provide a high-level overview of strategic learning across the health system (organizational, subnational, and national) to better understand how the concept of “learning systems” fits within this overall landscape. The second objective was to complete an in-depth review of the literature on “learning systems” by expanding on the definition provided by the Institute of Medicine and identifying common themes for building a continuous cycle of collaboration, feedback, and improvement across all levels of the health system.

## Methods

**Fig. 1:**
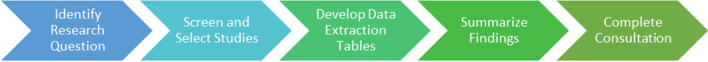
Scoping review framework

Scoping reviews are a common approach for clarifying definitions, mapping the key themes of a topic based on research evidence, and highlighting knowledge gaps in the literature [17]. This type of literature review was selected given the overall aim of this paper to explore guiding principles for strategic learning to improve the quality of health services across the health system. A protocol was developed based on the methodological framework from Arksey and O’Malley [18] and the Joanna Briggs Institute (JBI) Reviewer’s Manual for scoping reviews [19]. This was an iterative process that included an exploratory search for articles on learning systems using free-text terms in the JBI Database of Systematic Reviews and Implementation Reports, Cochrane Database of Systematic Reviews, and MEDLINE to develop, pilot, and refine the search strategy based on the aim of the scoping review [20]. The scoping review consisted of five stages, as outlined in Fig. 1: (1) developing the research question and search objectives based on the priority areas for the WHO GLL for Quality UHC and initial search results, (2) identifying the inclusion criteria, screening and selecting relevant studies, (3) extracting and analysing data from selected articles, (4) summarizing and reporting the findings, and (5) completing an online consultation with experts in healthcare improvement to obtain feedback on the recommendations.

### Research questions

The following research questions were explored in the literature.

#### Primary

What are the guiding principles for building a national learning system to inform policies and strategies for quality grounded in frontline realities?

#### Secondary

What are the levels for strategic learning across the health system focused on quality improvement (organizational, subnational, and national)? What are the common themes for forming a learning system to deliver quality health services?

### Search strategy

As recommended by JBI, the PCC (Population, Concept and Context) framework was used to define the search terms based on the research question and objectives of the scoping review. The PCC framework was also used to define the inclusion criteria for selecting relevant articles (Table 1). Four databases pertinent to the scoping review topic were searched on 17 June 2019 for publications between 2009 and 2019 (Global Health, MEDLINE, HealthSTAR, and CINAHL) using the search terms in the PCC framework as follows: (“Health” OR “Healthcare”) AND “Quality” AND (“Improvement” OR “Improvements”) AND “Learning”. A more conservative approach was used for the search terms to obtain a broader range of articles after piloting the initial search strategy. For example, the terms “health/healthcare” were used instead of “health system” to identify studies at multiple levels of the health system (e.g., health professionals/organizations). Similarly, the term “learning” was used on its own instead of “learning systems” to yield broader results that cover the topic of strategic learning in general across the health system, in addition to learning systems, and to account for the variability in terminology. Finally, the terms “quality” and “improvement” were selected to capture articles that addressed the continuous cycle of feedback and learning for the purposes of improving the delivery of quality health services.

**Table 1:** Search strategy

Objectives	Inclusion criteria	Search terms
*Learning levels* (High-level review): Identify the levels for strategic learning to deliver quality health services from a health system perspective *Learning systems* (Focused review): Identify common themes for building a learning system for delivering quality health services	*P (Population)* Any health condition/population	*P (Population)* Health/Healthcare
	*C (Concept)* Improving the quality of health services	*C (Concept)* Quality Improvement/Improvements
	*C (Context)* Collaborative learning at any level of the health system Any country/language Published between 2009 and 2019	*C (Context)* Learning

### Study selection

**Fig. 2:**
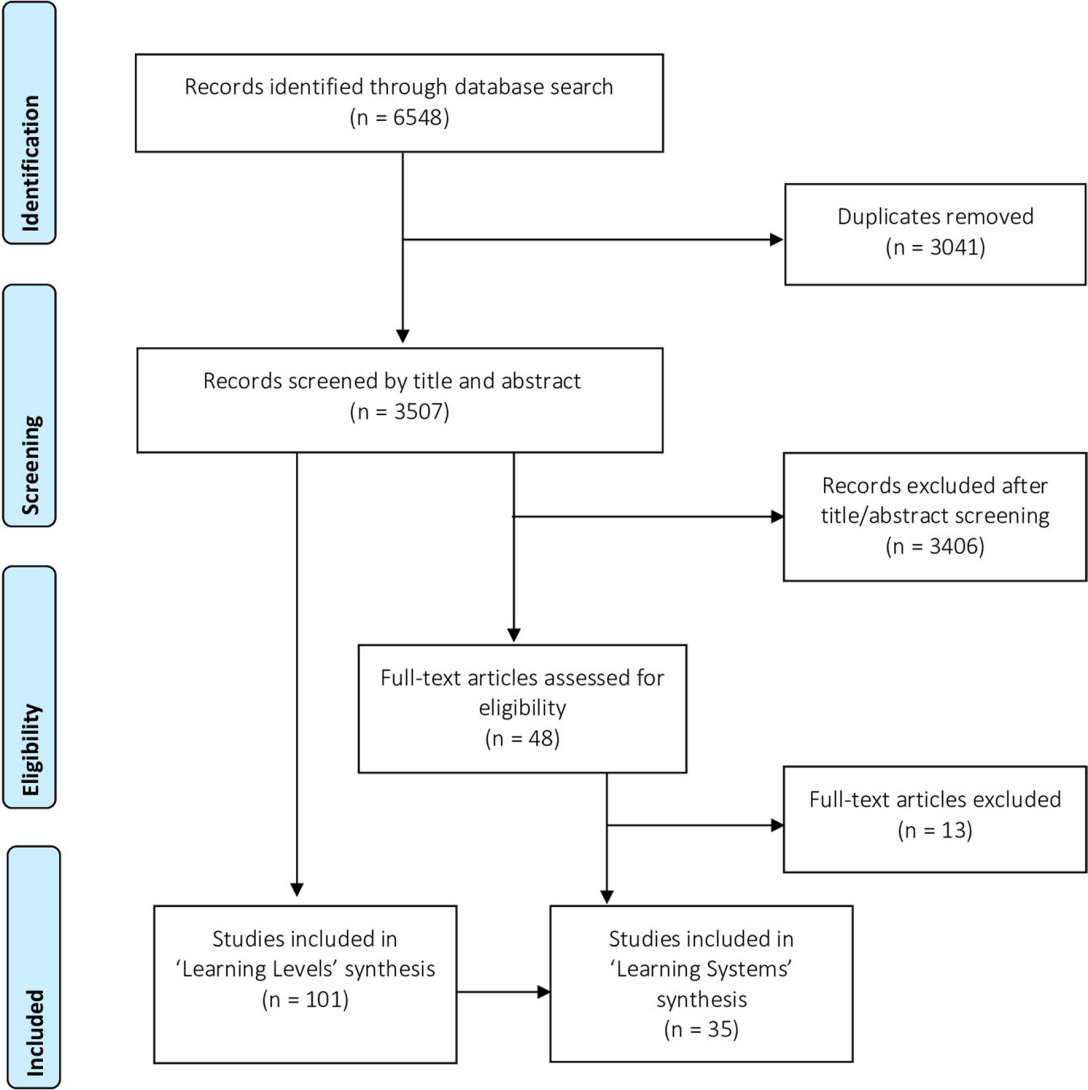
PRISMA study flow diagram

The search and screening results are reported in Fig. 2 using the PRISMA (Preferred Reporting Items for Systematic Reviews and Meta-Analysis) guidelines [21]. The search strategy resulted in 3507 records after the duplicates were removed. These records were screened using a two-step process based on the following inclusion criteria: (1) publications describing collaborative learning approaches to improve the delivery of health services from a system perspective (conceptually and/or empirically), (2) in the context of any health condition or healthcare setting, (3) at any level of the health system (organizational, subnational, and national), and (4) published between January 2009 and June 2019. Articles were excluded if the learning methods were unclear, the focus was on individual learning or learning technology (simulation, artificial intelligence, and complex data analysis), or the context of learning was outside the health sector. The articles were screened by titles and abstracts according to the inclusion criteria, where 101 articles were selected for the high-level “learning levels” synthesis. Of the 101 articles on “learning levels”, 48 records covered the topic of “learning systems” and were retained for a full-text review. Of the 48 records, 35 articles provided clear recommendations for building a learning system and were retained for the “learning systems” synthesis. Additional file 1 has the references of included articles by learning level, and Additional file 2 has the citations of excluded articles during the full-text review.

### Data analysis

The characteristics of each study were extracted and charted in a data extraction table that identified the title and authors, publication year, country, learning level to improve the quality of health services, and key findings. The authors met regularly to validate the data extraction and discuss the data analysis based on the priority areas for the WHO GLL for Quality UHC. A descriptive analysis was completed to define strategic learning at each level of the health system: “health professional level”, “organizational level”, “subnational/national level”, “multiple levels”, and “global level”. The characteristics of the learning levels were summarized in a table that included an amalgamated definition from a health system perspective, the number of articles, the list of countries, the types of settings, and the main topics (Additional file 3).

A thematic analysis was completed for articles on “learning systems” to identify common themes for building a continuous system of collaboration, feedback, and improvement across the health system. The full-text assessment was done independently by two reviewers, where the characteristics of each eligible study were presented in a summary table that included the title, the country, the main focus, the level of the health system (organizational, subnational, and national), and the definition of “learning system” and its key features (Additional file 4).

### Consultation

A summary of the scoping review results and recommendations was prepared for an online consultation with experts in healthcare improvement. The four experts referenced in the acknowledgements section of this paper were invited to answer open-ended questions on the definition of a learning system, the learning levels, and the guiding principles for forming a national learning system. They noted the importance of this work and that the nationwide framework provided a structure for quality improvement efforts across the health system. They also provided recommendations that complemented the findings from the scoping review. This included emphasizing the complexity of the health sector, the role of the public in setting priorities for the national learning system, and the importance of learning from unsuccessful quality improvement initiatives.

## Results

The first objective of the scoping review was to understand at a high level the levels for strategic learning to deliver quality services across the health system. Given the complexity of the health sector, it is important to have a global view of learning to understand the stakeholders involved in delivering quality health services and their role in a national learning system. A total of 101 articles met the inclusion criteria and were retained for the “learning levels” synthesis. All the articles were published in English. These articles were grouped into five categories: “health professional level” (19%), “health organizational level” (15%), “subnational/national level” (26%), “multiple levels” (35%), and “global level” (6%).^1^ The abstracts of these articles were reviewed to identify at a high level the characteristics of each learning level from a health system view. The full text of the article was reviewed when this information was not available in the abstract (or was unclear). The results are summarized in Additional file 3, including an amalgamated definition developed for learning at each level of the health system based on common trends.

The second objective of the scoping review was to complete a focused review of the literature on “learning systems” and identify common themes for building a continuous system of feedback, learning, and improvement across the health system. A total of 35 articles were retained for the “learning systems” synthesis. All the articles were published in English. Thirty-one articles were from high-income (HI) countries (89%) and four were from low- and middle-income countries (LMIC) (11%). The common objectives of the articles were to summarize the key features of a learning system to improve the quality of health services. Of the 35 articles, one was a quantitative study (3%), two were quality improvement reports (6%), and the other 32 were perspective/commentary papers (91%). Seven of the articles covered learning systems at the organizational level (20%), five at the subnational level (14%), 16 at the national level (46%), and one at the global level (3%). The other six articles did not have a defined scope for the learning system (17%). The results are summarized in Additional file 4.

### Health professional level

#### Definition

Health professionals are responsible for assessing, diagnosing, treating, and preventing health conditions based on the local needs of their community. Examples include medical doctors, nursing professionals, midwifery professionals, dentists, and pharmacists. Health professionals play an essential role in a national learning system by identifying and implementing improvements and recognizing systemic barriers to quality based on their frontline experiences [22, 23]. Health professionals need to be supported to develop the competencies required to lead changes at the point of care in a complex health system.

#### Strategic learning

Pre-service training in quality improvement is a pathway for building the competencies of future health professionals to continuously improve the quality of health services as part of their undergraduate and graduate training. Health professionals would benefit from early training to develop their knowledge, skills, and attitudes towards quality improvement and learning how to work as part of a broader interprofessional team within a larger health system.

#### Application

There is an increased focus on quality improvement training in medical and nursing education. Training methods address knowledge, skills, attitudes, and interprofessional collaboration in healthcare improvement. Training in practice-based learning and improvement helps trainees develop the competencies needed to continuously identify and implement improvements based on data in the service environment including hospital and primary care [24–30]. While students are trained in quality improvement methods to help them identify service improvement opportunities, a health system view is also needed to recognize systemic barriers as a limitation of improvement efforts [23, 31]. Systems-based practice requires trainees to have a broader understanding of the health system and associated challenges, and participate in system-level improvement initiatives [32, 33]. Combining practice-based learning and improvement with systems-based practice is an emerging concept to help prepare trainees to effectively function within a national learning system [34]. Interprofessional learning is another important theme in healthcare improvement where learning becomes more relationship-based and complex in the service environment [35–37]. Health professionals are expected to work as part of a team but are often trained in silos. More opportunities are needed for interprofessional learning to improve the quality of health services [38]. The role of pre-service training in quality improvement needs more attention in the literature [39, 40].

### Health organizational level

#### Definition

Health organizations are responsible for delivering quality health services to a defined community, including hospitals, primary care clinics, and community health centres. As the learning cycle in a national learning system begins with the interaction between the patient and the health professional, it is important to develop the characteristics needed to support learning and improvement at the organizational level [41].

#### Strategic learning

Participating in quality improvement initiatives is a pathway for a health organization to become a learning one by promoting accountability and building a culture of continuous feedback and improvement in the service environment based on successes and failures. Healthcare organizations are knowledge-intensive and rely on extensive professional experience, skills, and knowledge to be effective [42]. Having an organization-wide data-driven approach to patient care promotes continuous collaboration, learning, and improvement [43].

#### Application

A foundational characteristic of a learning organization is psychological safety, where people feel comfortable raising concerns and trying new approaches to improve health services and are accountable as a team for the results [41, 44–46]. Another important characteristic is interprofessional collaboration to improve communication, create positive learning experiences, and facilitate improvement [47–50]. Health professionals gain a deeper understanding of how their collective tasks are interdependent and impact the overall quality of health services [50]. Other common themes for promoting a culture of continuous collaboration, learning, and improvement include leadership commitment, creative problem-solving, and performance measurement [42, 51–54]. The digitization of patient care facilitates real-time sharing of data and generates new knowledge for learning and improvement [55].

Health organizations are characterized by people, knowledge, and information technology, and are key components of an effective national learning system. The focus of health organizations is on identifying and addressing organization-specific areas for improvement. However, there are mutual benefits for adopting a health system perspective and exchanging learnings with other organizations to leverage experiences and spread improvements for common challenges.

### Subnational/national level

#### Definition

Multiple health organizations overseen by health authorities at the subnational or national levels to guide the delivery of health services through supportive policies and strategies on quality. The specific roles will vary depending on the structure of a country’s health system.

#### Strategic learning

Learning collaboratives are a pathway for multiple health organizations within a subnational or national health system to become a learning one by participating in quality improvement initiatives. These are temporary initiatives where a group of healthcare organizations learn together to improve a common priority area generally at the subnational or national level. The scoping review focused on learning collaboratives that leveraged opportunities for group learning. As healthcare is a complex adaptive system, it is important to study not only whether a quality improvement initiative works within a collaborative, but also how and why [56].

#### Application

Learning collaboratives are generally short-term initiatives that bring together healthcare teams to spread a specific improvement by applying a change package of interventions. Learning collaboratives covered various clinical settings including primary care, community care, acute care, and specialized services. They were used by HI and LMIC. Models for improvement generally included an aim statement, a project team, team-based learning using training materials (e.g., change package and online platform) and activities (e.g., in-person workshop and coaching), and performance evaluation. The Institute for Healthcare Improvement Breakthrough Series collaboratives were referenced as an evidence-based model for improvement through several studies. Topics ranged from adherence to specific clinical practice guidelines for chronic diseases (e.g., diabetes and obesity) to broader improvements to integrated care (e.g., improving mental health services in primary care). One study conducted a series of mini-collaboratives to build quality improvement capacity across several states and prepare for national accreditation [57].

Successful collaboratives included leadership support, clear objectives and time frames, opportunities for peer-to-peer learning, evidence-based improvements, psychological safety, and development of transferrable competency in quality improvement [56–65]. Learning collaboratives are temporary in nature and seem to be effective for single-target interventions [66–70]. However, practice transformation such as integration of care is complex and requires a multilevel approach to achieve improvement and adaptation to the local context [71–74]. More complex learning collaboratives provided opportunities to improve interorganizational communication, and connections between services where health professionals view themselves as part of a larger system of care [63, 75–77]. Learning collaboratives also provided opportunities to build relationships, create networks of experts to exchange best practices, and improve implementation of quality improvement initiatives [78–80].

Barriers included protected time to participate in the collaborative, few opportunities to co-design the collaborative, lack of information sharing and coordination of care between sectors, and lack of data on the sustainability of the results due to the temporary nature of the collaborative [58, 81, 82]. The evaluation tends to focus more on the outcomes rather than the learning methodology and associated costs.

While learning collaboratives have more of a subnational/national health system perspective towards learning by broadening the reach of improvements, they do not address systemic barriers that are outside the control of participating organizations because they tend not to involve subnational/national health authorities as stakeholders.

### Multiple levels

#### Definition

Quality improvement initiatives involving multiple stakeholders learning together to continuously improve the quality of health services based on the frontline experience.

#### Strategic learning

A learning system is formed by a group of stakeholders operating at an organizational, subnational, national, or global level to continuously improve the quality of health services based on real-world evidence. The purpose of the learning system is to provide the best care at the lowest cost by having researchers partner with patients, families, practitioners, and other stakeholders to co-create knowledge based on real-world evidence and promote mutual learning for improving the quality of health services and ensuring integrated patient-centred care [83–86]. Additional file 4 references the definitions found in the literature for learning systems, where several studies have used or adapted the definition provided by the Institute of Medicine [87].

#### Application

“Learning systems” is an emerging concept that was first formally defined by the Institute of Medicine in 2007 [87]. Therefore, there is some, albeit limited, evidence on the positive impact of learning systems on patient outcomes [85, 88, 89]. However, there is guidance on the core features of learning systems and the process used to develop them based on examples from both HI and LMIC.

The first common feature of a learning system is designating a *network of stakeholders* responsible for the design, operation, and governance of the system, including establishing a model for improvement and fostering a collaborative environment. Stakeholders include patients, families, health professionals, administrators, researchers, and policy-makers [90–95]. Learning is needed at multiple levels of the health system, from practice to policy, to evaluate the quality of health services at the population level [96]. It is recommended to start with a prototype for a learning system that could be replicated by other areas in healthcare and gradually scale to the national or global level based on continuous learning and improvement [86, 94, 97, 98].

The second feature of a learning system is having a *common goal* and commitment to improving one or more areas in the delivery of health services. This shared purpose fosters a sense of community among participants of the learning system where collaboration is a fundamental requirement for learning [84, 85, 94, 98–101].

The learning cycle starts with the patient–clinician interaction at the point of care [102]. The third feature of a learning system is generating *standardized approaches to care and quality measures* based on patient data collected at the point of care combined with research and expertise [103, 104]. Quality measures include processes of care, patient experience, and patient outcomes. They are used to test new ideas, in assessing performance against best practices, and for benchmarking and improvement across the health system [104–106]. Quality measures and incentives are needed to encourage continuous learning and improvement and achievement of common quality goals [83, 105, 107–109].

The fourth feature of a learning system is *leveraging technology* (e.g., electronic health records and patient registries, and virtual platforms) to collect “real-world evidence” at the point of care, provide real-time access to knowledge including clinical decision support tools and treatment options, and facilitate the flow of knowledge between the participants of the learning system [94, 104, 108, 110–114].

The fifth feature of a learning system is building *trust, transparency, and accountability* among all stakeholders. Public reporting is required to share knowledge on the quality of health services with the public and other stakeholders, including national or subnational comparisons and benchmarks. Transparency helps build trust and accountability and incentivizes learning and improvement [105, 107, 109, 115]. It is important that a learning system is designed in a way that protects the privacy, confidentiality, and security of patient data [92, 93, 95].

Learning relies on trust and collaboration. One of the challenges with a learning system is fostering a sense of community with mutual benefits within a fragmented health system [116]. When health services are organized and funded in silos, this creates misaligned interests and does not reflect the path of the patient who needs an integrated health system. Learning systems for military health services have demonstrated that having an integrated health system promotes aligned interests and facilitates the flow of information within the learning system [109, 117]. It is recommended that the health system be designed in a way that promotes learning from the beginning, specifically in LMIC, to avoid retrofitting the health system at a later stage [91].

Learning systems serve similar needs in HI and LMIC, which are to ensure the delivery of quality health services based on continuous learning and improvement. Poor quality of care is one of the leading causes of mortality in LMIC, and contributed to five million deaths based on data from the 2016 Global Burden of Disease study [118]. However, additional challenges are noted for LMIC, including limited resources to electronically collect data at the point of care and evaluate the impact of quality improvement initiatives on patient outcomes [119]. There is also little standardization of patient health records and hospital forms, which makes the use of routine clinical data a challenge [90]. Another challenge is translating evidence into practice. Quality improvement initiatives in LMIC tend to be externally led, with limited insight into contextual barriers and/or lacking local evidence. It is important that learning systems include representation from interprofessional teams working at the point of care and that initiatives are adapted to the local context [105, 119].

### Global level

There is potential to form a network with other learning systems globally around a common, complex healthcare challenge to foster cross-learning between countries as they build or strengthen their learning systems. A learning network is a group of learning systems operating at a national or global scale for mutual learning and improvement on a common priority theme. Learning networks rely on collaboration and a bottom-up approach to learning and improvement [120–122]. The concept of a “learning network” was used to refer to either learning collaboratives or learning systems [122, 123]. However, learning networks could also be defined as a method for bringing learning systems together around a common theme such as primary care [86, 124, 125]. There are few studies in the literature on learning networks, in particular on a global scale.

## Discussion

A health system perspective is needed to continuously improve the quality of health services at the point of care while simultaneously addressing systemic barriers. Having a health system perspective of learning and improvement means recognizing that health professionals are part of an interdisciplinary team within a health organization that is part of a subnational/national health system that is accountable to patients, families, and the broader community [83, 98]. While it is unclear whether one quality improvement model is more effective than another, some of the success factors seem to include having a systemwide approach that considers the local context and involves multiple stakeholders learning together to achieve improvement based on a systematic review of quality improvement models [86, 126, 127]. A national learning system involves stakeholders across the health system and uses a collaborative process of translating patient data into knowledge that can be used to make changes within and beyond health organizations to improve the quality of health services through supportive strategies and policies grounded in the frontline experiences of patients, family members, and health professionals [96].

### A global compass for national learning systems

Dr. Don Berwick highlights the fact that “more than ever before, [we need] a system devoted to continual learning and improvement of patient care, top to bottom and end to end” [128]. This paper recommends guiding principles for transforming a country’s health system into a national learning system based on the scoping review results and consultation with experts in the field. The premise of this approach is that the national learning system is built from the ground up based on frontline experiences of patients, families, and health professionals and the broader needs of the community to guide a national strategy for quality health services [88, 89, 102].

The compass of the national learning system is centred on four cross-cutting themes across the health system: alignment of priorities, systemwide collaboration, transparency and accountability, and knowledge sharing of real-world evidence generated at the point of care.

**Fig. 3:**
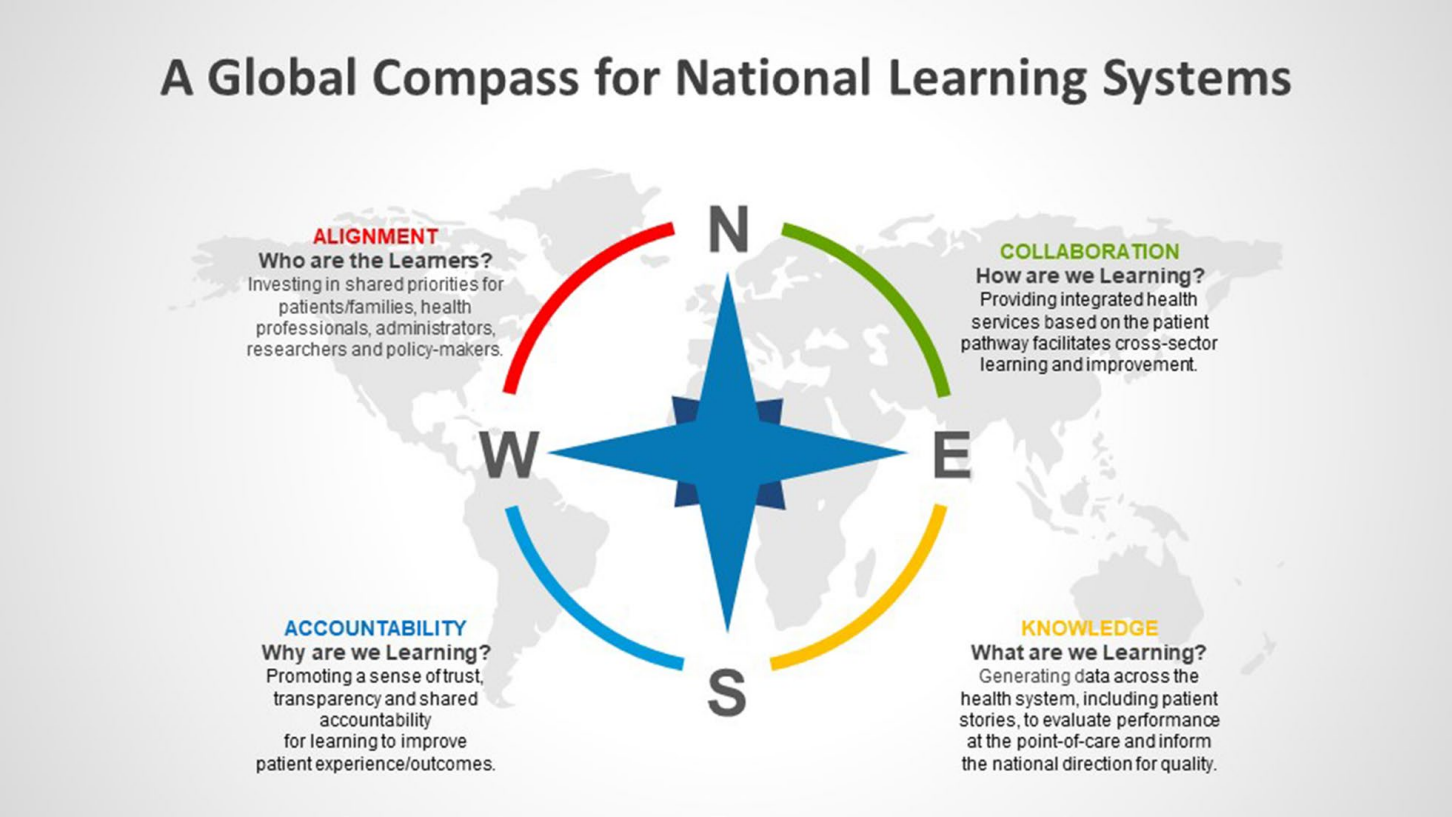
Global compass for national learning systems

The following is a summary of these four themes illustrated in Fig. 3, where a governance model is needed to set the stage for learning at the national level to facilitate the flow of information across the various levels of the health system [91, 95, 105]. The multiple arrows illustrate how the flow of learning and improvement across the entire health system can influence direction at all levels (top-down, bottom-up, and end to end).

#### Alignment: Who are the learners?

Investing in shared priorities for patients, families, health professionals, administrators, researchers, and policy-makers facilitates the uptake of best practices at the point of care [84, 90, 101, 117]. Priorities should be in line with the overall national direction for quality health services [98, 105]. This helps standardize the delivery of quality health services at the subnational level (e.g., district, regional, or county) and minimize variations as much as possible. Adaptations may be needed at the organizational level based on the local context [96, 103, 110].

#### Collaboration: How are we learning?

Providing integrated health services based on the patient pathway facilitates cross-sector learning and improvement. This includes the spread of best practices to other organizations at the subnational level (or other districts), and interprofessional collaboration and patient/community engagement to harness learnings and improvements at the organizational level [86, 99, 101]. Examples of patient/community engagement include participating in advisory committees to inform quality policies/strategies, research design, and implementation of quality improvement initiatives [94, 117].

#### Accountability: Why are we learning?

Promoting a sense of trust, transparency, and shared accountability for learning and improvement across the health system includes creating financial incentives to focus on patient experience/outcomes, publicly sharing reports on the health system’s performance, and having a safe space for health professionals to share variations from best practices and/or lessons learned [92, 100, 105, 115, 116].

#### Knowledge: What are we learning?

Training on “systems thinking” established as part of medical education can support health professionals to recognize systemic barriers based on their frontline experience [34, 111]. Data are generated across all levels of the health system, including patient stories, to evaluate performance at the point of care (including costs), strengthen the health system to inform service planning efforts at the subnational level, and inform the national quality strategy [90, 91, 108, 109, 117].

### National learning system levels

As the learning cycle begins with the interaction between patients and health professionals, the perspectives of these stakeholders are essential to guide the national direction on quality, the systemwide implementation of quality improvement initiatives, and the delivery of quality health services at the point of care [88, 89, 102]. Therefore, it is important to prepare health professionals and health organizations for their role within a national learning system. Health professionals can develop competencies in quality improvement and help identify systemic barriers to delivering quality health services [32–34]. Health organizations can become learning organizations by participating in quality improvement initiatives, including learning collaboratives, to cultivate a culture of continuous improvement, patient partnership and community engagement, and shared learning [42, 43].

**Fig. 4:**
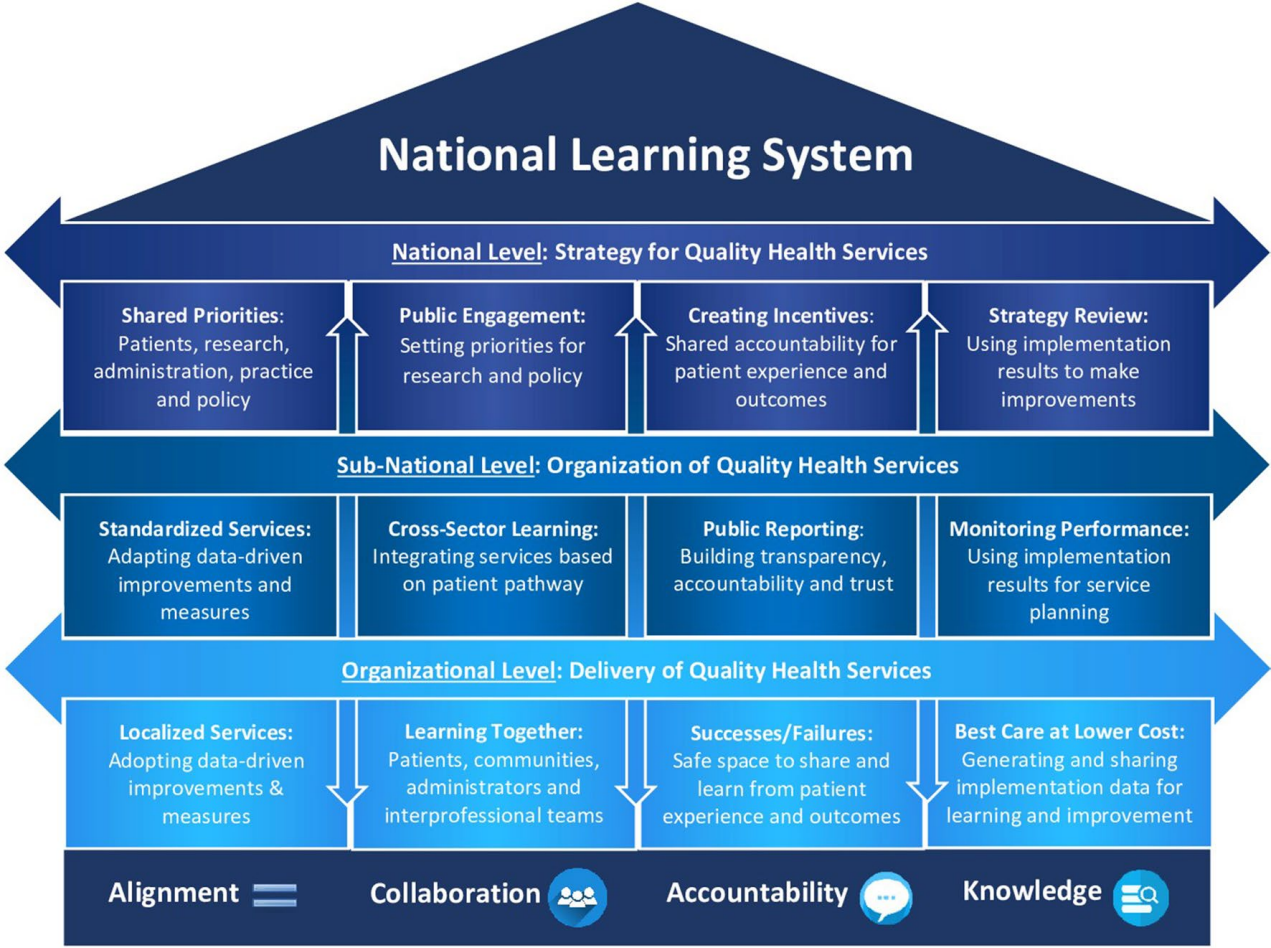
National learning system to improve the quality of health services

The following is an overview of the guiding principles for learning and improvement at each level of the health system illustrated in Fig. 4. It is recommended to start with a prototype for a learning system in a specific health priority area and gradually scale to the national level [94]. An example is provided for illustrative purposes on how to apply these guiding principles to mental health services in primary care [129].

#### Health organizational level: delivery of quality health services

As a national learning system is built from the ground up based on the frontline experiences of patients, families, and health professionals, stakeholder input is obtained from health organizations to support adherence to evidence-based guidelines and data collection requirements, team-based approach to quality health services, shared learning, and measurement [88, 103]. For example, in a national learning system focused on quality mental health services in primary care, the following interventions could be identified at the organizational level to achieve quality integrated care: patient registry, collaboration between primary care and mental health clinicians including ongoing mentorship and training, adherence to evidence-based guidelines with routine screening and active follow-up to support treatment adherence, comprehensive treatment options including evidence-based psychotherapy and/or medications, stepped approach to care based on patient’s response to treatment, and leveraging patient data in electronic health records to learn from patient outcomes [129].

#### Subnational level: organization of quality health services

In a national learning system framework for improving the quality of health services, the organization of quality health services at the subnational level involves standardizing the delivery of health services, integrating health services based on the patient pathway, reporting performance results to the public, and monitoring performance and using the results to make improvements [97, 107]. In the example of a national learning system for mental health services in primary care, the subnational health system would be responsible for the systemwide implementation of integrated care for mental health services in primary care clinics within its jurisdiction. Examples of interventions at the subnational level to achieve quality integrated care include the following: standards and measures for mental health services in primary care clinics, publicly funded treatment options based on evidence with costs tracked, electronic health record database with patient data documented across the integrated care pathway from screening to follow-up visits, primary care clinics incentivized to provide integrated plans in consultation with mental health clinicians, routine screening policy informed by electronic health record data, and reporting of patient outcomes for mental health services in quality improvement plans at the subnational health system level [129].

#### National level: strategy for quality health services

In a national learning system for improving the quality of health services, the strategy for quality health services is overseen at the national health policy level, including identifying and setting priorities for public policy, incentivizing a collaborative focus on patient experiences and outcomes, and using frontline data to review and improve the strategy [90, 95, 98, 109]. In a national learning system for mental health services in primary care, the following interventions could be identified at the national level to support quality integrated care: national guidelines for mental health services in primary care, policy on public coverage of evidence-based treatments, national electronic health record database for primary care clinics, action plan to support the integration of mental health professionals in primary care, and screening recommendations [129].

### Future directions

There is a gap in the literature on how to operationalize learning systems, specifically at the national level. The proposed guiding principles for building a national learning system is the first step in learning to develop, implement, and refine national policies and strategies for quality based on the frontline experiences of patients, families, and health professionals. More research is required to evaluate the impact of a national learning system on patient outcomes. The national learning system will need to be applied, evaluated, and reviewed based on implementation results. As national health systems continue to face common challenges, there are opportunities to exchange learnings with other countries by forming a global network of learning systems.

Possible applications of the national learning system depend on country priorities. However, a common challenge is for countries to refocus their current model to invest in prevention and quality health services in the community. An example would be building a national learning system to understand the policies and strategies needed to improve the quality of mental health services in primary care.

## Strengths and limitations

The first limitation of this scoping review is that the articles were screened by one reviewer. However, two reviewers independently completed the full-text review and extracted the relevant data from the articles. The data summary tables were also shared with the authorship team for validation against the research question and objectives of the scoping review and the inclusion criteria. The second limitation is the adaptation of the methodological framework for scoping studies from Arksey and O’Malley based on the objectives of this scoping review. The learning levels for improving the quality of health services were identified by reviewing the abstracts to understand their respective role in building a national learning system. The full text was reviewed when this information was not available in the abstract (or was unclear).

The high-level review of all the “learning levels” provided a more holistic view of learning to improve the quality of health services and complemented the detailed review of the “learning systems” category by allowing for richer recommendations. Another strength of this scoping review is the consultation with experts in healthcare improvement to complement the results and recommendations based on expertise and experience.

## Conclusion

“Every system is perfectly designed to get the results it gets” is a popular quotation in the world of healthcare improvement [130]. Government health authorities need to consider lessons from the point of care when setting their national direction on quality health services. Therefore, it is important to understand the layers for strategic learning across the health system. Building a national system of learning and improvement requires shared priorities, collaboration, public trust and accountability, and knowledge sharing within and between countries. This paper proposes guiding principles for building a national learning system that bridges the gap between practice and policy based on the needs of patients, families, and the broader community. Future research is needed to validate the application of this approach, adapt the guiding principles to a country’s context and priorities, and make improvements based on the findings.

### Key messages

- There is an urgent need to understand how different health systems are developing the learning architecture required to implement national strategic direction on quality health services.
- This scoping review provides granular-level information that can be considered by those responsible for developing a learning architecture for quality health services.
- This scoping review has led to an approach to building a national learning system that requires shared priorities, collaboration, public trust and accountability, and knowledge sharing within and between countries.
- Future research is needed to validate the application of this approach and make improvements based on the findings.

## Supplementary Information

**Additional file 1.** References of articles included by “Learning Level”.

**Additional file 2.** References of articles excluded during full-text review.

**Additional file 3.** Summary of studies included by “Learning Level”.

**Additional file 4.** Characteristics of studies included for the “Learning Systems” synthesis.

## Data Availability

Available in additional documents.

## Abbreviations

UHC: Universal health coverage
GLL: Global Learning Laboratory
JBI: Joanna Briggs Institute
PCC: Population, Concept and Context
PRISMA: Preferred Reporting Items for Systematic Reviews and Meta-Analyses
HI: High-income
LMIC: Low- and middle-income countries

## Footnotes

1 Percentages may not add up to exactly 100% due to rounding.
