# Supplementary material for: Aiming for quality: a global compass for national learning systems
Source: Health Res Policy Syst. 2021 Jul 19;19:102. doi: 10.1186/s12961-021-00746-6 (PMC8287697; doi:10.1186/s12961-021-00746-6)
Supplement: Supplementary file 1 — Additional file 1. References of articles included by “Learning Level”. [file 12961_2021_746_MOESM1_ESM.docx]

**Additional file 1: References of Articles Included by “Learning Level”**

| Category | Reference |
| --- | --- |
| Health Professional Level | Anagnostou, V.K., et al., A longitudinal, collaborative, practice-based learning and improvement model to improve post-discharge heart failure outcomes. Connecticut Medicine, 2014. 78(1): p. 33-6.  Butler, J.M., et al., "It Feels Like a Lot of Extra Work": Resident Attitudes About Quality Improvement and Implications for an Effective Learning Health Care System. Academic Medicine, 2017. 92(7): p. 984-990.  Chen, C.A., et al., How we used a patient visit tracker tool to advance experiential learning in systems-based practice and quality improvement in a medical student clinic. Medical Teacher, 2016. 38(1): p. 36-40.  Fowler, T.O., et al., Alignment of an interprofessional student learning experience with a hospital quality improvement initiative. Journal of interprofessional care, 2018(9205811): p. 1-10.  Huntington, J.T., et al., A standardized curriculum to introduce novice health professional students to practice-based learning and improvement: a multi-institutional pilot study. Quality Management in Health Care, 2009. 18(3): p. 174-81.  Kataoka, S.H., et al., MAP as a model for practice-based learning and improvement in child psychiatry training. Journal of Clinical Child & Adolescent Psychology, 2014. 43(2): p. 312-22.  Kenaszchuk, C., et al., Positive and null effects of interprofessional education on attitudes toward interprofessional learning and collaboration. Advances in Health Sciences Education, 2012. 17(5): p. 651-69.  O'Connor, E.S., et al., Developing a practice-based learning and improvement curriculum for an academic general surgery residency. Journal of the American College of Surgeons, 2010. 210(4): p. 411-7.  Pensa, M., P. Frew, and S.B. Gelmon, Integrating improvement learning into a family medicine residency curriculum. Family Medicine, 2013. 45(6): p. 409-16.  Portney, L., A.F. Johnson, and M. Knab, Preparing Future Health Professionals for Interprofessional Collaborative Practice Part 1: The Context for Learning. Seminars in Speech & Language, 2017. 38(5): p. 335-341.  Ross, A.M., Learning about practice-based evidence in outcomes management. Communicating Nursing Research, 2010. 43: p. 165-165.  Smith, K.L., et al., Residents contributing to inpatient quality: blending learning and improvement. Journal of Hospital Medicine (Online), 2012. 7(2): p. 148-53.  Tomolo, A.M., et al., Pilot study evaluating a practice-based learning and improvement curriculum focusing on the development of system-level quality improvement skills. Journal of graduate medical education, 2011. 3(1): p. 49-58.  Wehbe-Janek, H., et al., Preparing Academic Medical Centers for the Clinical Learning Environment Review: Alliance of Independent Academic Medical Centers National Initiative IV Outcomes and Evaluation. The Ochsner journal, 2016. 16(2): p. 166-71.  Wittich, C.M., et al., Perspective: Transformative learning: a framework using critical reflection to link the improvement competencies in graduate medical education. Academic Medicine, 2010. 85(11): p. 1790-3.  Wong, A.K.C., et al., The effect of interprofessional team-based learning among nursing students: A quasi-experimental study. Nurse Education Today, 2017. 53(ned, 8511379): p. 13-18.  Wysham, N.G., et al., Development and Refinement of a Learning Health Systems Training Program. EGEMS (Washington, DC), 2016. 4(1): p. 1236.  Yang, Y.-Y., et al., A model of four hierarchical levels to train Chinese residents' teaching skills for "practice-based learning and improvement" competency. Postgraduate Medicine, 2015. 127(7): p. 744-51.  Yazdani, S., A. Farajpour, and S. Shakerian, Practice-based learning and improvement (PBLI) from the perspective of Iranian medical education experts: a thematic content analysis. Iranian Red Crescent Medical Journal, 2017. 19(7): p. e55664. |
| Health Organizational Level | Albritton, J.A., et al., The role of psychological safety and learning behavior in the development of effective quality improvement teams in Ghana: an observational study. BMC health services research, 2019. 19(1): p. 385.  Bunniss, S., F. Gray, and D. Kelly, Collective learning, change and improvement in health care: trialling a facilitated learning initiative with general practice teams. Journal of Evaluation in Clinical Practice, 2012. 18(3): p. 630-6.  Hovlid, E., et al., Sustainability of healthcare improvement: what can we learn from learning theory? BMC Health Services Research, 2012. 12(101088677): p. 235.  Liu, V.X., et al., Data that drive: Closing the loop in the learning hospital system. Journal of Hospital Medicine, 2016. 11: p. S11-S17.  Lyman, B., et al., Organizational Learning in a Cardiac Intensive Care Unit. Dimensions of Critical Care Nursing, 2017. 36(2): p. 78-86.  Lyman, B., et al., Organizational Learning in Hospitals: A Realist Review. Journal of advanced nursing, 2019(7609811, h3l).  McNamara, D.A., P. Rafferty, and F. Fitzpatrick, An improvement model to optimise hospital interdisciplinary learning. International Journal of Health Care Quality Assurance, 2016. 29(5): p. 550-8.  Nembhard, I.M. and A.L. Tucker, Applying Organizational Learning Research to Accountable Care Organizations. Medical Care Research & Review, 2016. 73(6): p. 673-684.  Nembhard, I.M., P. Cherian, and E.H. Bradley, Deliberate learning in health care: the effect of importing best practices and creative problem solving on hospital performance improvement. Medical Care Research & Review, 2014. 71(5): p. 450-71.  Nystrom, M., Characteristics of health care organizations associated with learning and development: lessons from a pilot study. Quality Management in Health Care, 2009. 18(4): p. 285-94.  Nystrom, M.E., et al., Exploring the potential of a multi-level approach to improve capability for continuous organizational improvement and learning in a Swedish healthcare region. BMC Health Services Research, 2018. 18(1): p. 376.  Ortega, A., et al., Enhancing team learning in nursing teams through beliefs about interpersonal context. Journal of Advanced Nursing (John Wiley & Sons, Inc.), 2013. 69(1): p. 102-111.  Schilling, L., et al., Kaiser Permanente's performance improvement system, Part 4: Creating a learning organization. Joint Commission Journal on Quality & Patient Safety, 2011. 37(12): p. 532-43.  Shamshirsaz, A.A., et al., Multidisciplinary team learning in the management of the morbidly adherent placenta: outcome improvements over time. American Journal of Obstetrics & Gynecology, 2017. 216(6): p. 612.e1-612.e5.  Soklaridis, S., Improving hospital care: are learning organizations the answer? Journal of Health Organization & Management, 2014. 28(6): p. 830-8. |
| Sub-National/National Level | Baum, R.A., M.A. King, and L.S. Wissow, Outcomes of a Statewide Learning Collaborative to Implement Mental Health Services in Pediatric Primary Care. Psychiatric services (Washington, D.C.), 2019. 70(2): p. 123-129.  Beitsch, L.M., A. Pattnaik, and K. Madamala, The Multi-state Learning Collaborative storyboards: quality improvement lessons learned from 162 projects. Florida Public Health Review, 2013. 10(Florida State University College of Medicine, Center for Medicine and Public  Health, Tallahassee, Florida, USA.): p. 25-34.  Beitsch, L.M., et al., The quantitative story behind the quality improvement storyboards: a synthesis of quality improvement projects conducted by the Multi-State Learning Collaborative. Journal of Public Health Management and Practice, 2013. 19(4): p. 330-340.  Bunger, A.C., et al., Advice-seeking during implementation: a network study of clinicians participating in a learning collaborative. Implementation Science, 2018. 13(1): p. 101.  Cleary, S., et al., The everyday practice of supporting health system development: learning from how an externally-led intervention was implemented in Mozambique. Health Policy & Planning, 2018. 33(7): p. 801-810.  Freney, E., D. Johnson, and I. Knox, Promoting breastfeeding-friendly hospital practices: a Washington State learning collaborative case study. Journal of Human Lactation, 2016. 32(2): p. 355-360.  Gerrish, K., C. Keen, and J. Palfreyman, Learning from a clinical microsystems quality improvement initiative to promote integrated care across a falls care pathway. Primary health care research & development, 2018(100897390): p. 1-6.  Gillespie, S.M., et al., Pioneering a Nursing Home Quality Improvement Learning Collaborative: A Case Study of Method and Lessons Learned. Journal of the American Medical Directors Association, 2016. 17(2): p. 136-41.  Godfrey, M.M. and B.J. Oliver, Accelerating the rate of improvement in cystic fibrosis care: contributions and insights of the learning and leadership collaborative. BMJ Quality & Safety, 2014. 23 Suppl 1(101546984): p. i23-i32.  Hirschhorn, L.R., et al., Learning before leaping: integration of an adaptive study design process prior to initiation of BetterBirth, a large-scale randomized controlled trial in Uttar Pradesh, India. Implementation Science, 2015. 10(101258411): p. 117.  Kotecha, J., et al., Influence of a quality improvement learning collaborative program on team functioning in primary healthcare. Families, Systems, & Health, 2015. 33(3): p. 222-30.  Lipman, P.D. and C.B. Aspy, Local Learning Collaboratives to Improve Quality for Chronic Kidney Disease (CKD): From Four Regional Practice-based Research Networks (PBRNs). Journal of the American Board of Family Medicine: JABFM, 2016. 29(5): p. 543-52.  McHugh, M., et al., Changes in patient flow among five hospitals participating in a learning collaborative. Journal for Healthcare Quality, 2013. 35(1): p. 21-9.  Nadeem, E., et al., A literature review of learning collaboratives in mental health care: used but untested. Psychiatric Services, 2014. 65(9): p. 1088-99.  Nembhard, I.M., All teach, all learn, all improve?: the role of interorganizational learning in quality improvement collaboratives. Health Care Management Review, 2012. 37(2): p. 154-64.  Nembhard, I.M., Learning and improving in quality improvement collaboratives: which collaborative features do participants value most? Health Services Research, 2009. 44(2 Pt 1): p. 359-78.  Nordstrom, B.R., et al., Using a Learning Collaborative Strategy With Office-based Practices to Increase Access and Improve Quality of Care for Patients With Opioid Use Disorders. Journal of Addiction Medicine, 2016. 10(2): p. 117-23.  Okafor, M., et al., Explication of a Behavioral Health-Primary Care Integration Learning Collaborative and Its Quality Improvement Implications. Community mental health journal, 2018. 54(8): p. 1109-1115.  Prabhakaran, S., J. Lee, and K. O'Neill, Regional Learning Collaboratives Produce Rapid and Sustainable Improvements in Stroke Thrombolysis Times. Circulation. Cardiovascular Quality & Outcomes, 2016. 9(5): p. 585-92.  Rand, C.M., et al., A learning collaborative model to improve human papillomavirus vaccination rates in primary care. Academic Pediatrics, 2018. 18(2 Suppl.): p. S46-S52.  Russ, S.A., et al., Improving follow-up to newborn hearing screening: a learning-collaborative experience. Pediatrics, 2010. 126(Supplement 1): p. S59-S69.  Shaw, E.K., et al., Effects of facilitated team meetings and learning collaboratives on colorectal cancer screening rates in primary care practices: a cluster randomized trial. Annals of Family Medicine, 2013. 11(3): p. 220-8.  Shellhaas, C., et al., The Ohio Gestational Diabetes Postpartum Care Learning Collaborative: Development of a Quality Improvement Initiative to Improve Systems of Care for Women. Maternal & Child Health Journal, 2016. 20: p. 71-80.  Starkey, M., D. Wiest, and A. Qaseem, Improving Depression Care Through an Online Learning Collaborative. American Journal of Medical Quality, 2016. 31(2): p. 111-7.  Vannoy, S.D., et al., A learning collaborative of CMHCs and CHCs to support integration of behavioral health and general medical care. Psychiatric Services, 2011. 62(7): p. 753-8.  Werdenberg, J., et al., Successful implementation of a combined learning collaborative and mentoring intervention to improve neonatal quality of care in rural Rwanda. BMC Health Services Research, 2018. 18(941). |
| Multiple Levels | Atkins, D., A.M. Kilbourne, and D. Shulkin, Moving from discovery to system-wide change: the role of research in a learning health care system: experience from three decades of health systems research in the Veterans Health Administration. Annual Review of Public Health, 2017. 38(Veterans Health Administration, US Department of Veterans Affairs, Washington, DC 20420, USA.): p. 467-487.  Bailie, R., et al., A systems-based partnership learning model for strengthening primary healthcare. Implementation Science, 2013. 8(101258411): p. 143.  Beane, A., et al., A learning health systems approach to improving the quality of care for patients in South Asia. Global health action, 2019. 12(1): p. 1587893.  Bernstein, J.A., et al., Ensuring public health's future in a national-scale learning health system. American Journal of Preventive Medicine, 2015. 48(4): p. 480-7.  Bindman, A.B., The Agency for Healthcare Research and Quality and the Development of a Learning Health Care System. JAMA Internal Medicine, 2017. 177(7): p. 909-910.  Britto, M.T., et al., Using a network organisational architecture to support the development of Learning Healthcare Systems. BMJ quality & safety, 2018. 27(11): p. 937-946.  Brooks, D., et al., Developing a framework for integrating health equity into the learning health system. Learning health systems, 2017. 1(3).  Deans, K.J., S. Sabihi, and C.B. Forrest, Learning health systems. Seminars in Pediatric Surgery, 2018. 27(6): p. 375-378.  English, M., et al., Building Learning Health Systems to Accelerate Research and Improve Outcomes of Clinical Care in Low- and Middle-Income Countries. PLoS medicine, 2016. 13(4): p. e1001991-e1001991.  Etheredge, L.M., Rapid Learning: A Breakthrough Agenda. Health Affairs, 2014. 33(7): p. 1155-1162.  Forrest, C.B., et al., PEDSnet: a National Pediatric Learning Health System. Journal of the American Medical Informatics Association, 2014. 21(4): p. 602-6.  Forrest, C.B., et al., PEDSnet: how a prototype pediatric learning health system is being expanded into a national network. Health Affairs, 2014. 33(7): p. 1171-1177.  Hagland, M., lOM Report: 'The Path to Continuously Learning Healthcare in America'...Paul Tang, M.D. Healthcare Informatics, 2012. 29(9): p. 30-33.  Irimu, G., et al., Approaching quality improvement at scale: a learning health system approach in Kenya. Archives of disease in childhood, 2018. 103(11): p. 1013-1019.  Jiang, L., et al., Achieving best outcomes for patients with cardiovascular disease in China by enhancing the quality of medical care and establishing a learning health-care system. Lancet, 2015. 386 North American Edition(10002): p. 1493-1505.  Johnson, L.C., et al., Fostering Collaboration Through Creation of an IBD Learning Health System. American Journal of Gastroenterology, 2017. 112(3): p. 406-408.  Kamal, A.H., et al., A Person-Centered, Registry-Based Learning Health System for Palliative Care: A Path to Coproducing Better Outcomes, Experience, Value, and Science. Journal of palliative medicine, 2018. 21(S2): p. S61-S67.  Kilbourne, A.M., et al., Accelerating Research Impact in a Learning Health Care System: VA's Quality Enhancement Research Initiative in the Choice Act Era. Medical Care, 2017. 55 Suppl 7 Suppl 1(0230027, lsm): p. S4-S12.  Lowes, L.P., et al., 'Learn From Every Patient': implementation and early results of a learning health system. Developmental Medicine & Child Neurology, 2017. 59(2): p. 183-191.  Mason, A.R. and A.J. Barton, The emergence of a learning healthcare system. Clinical Nurse Specialist: The Journal for Advanced Nursing Practice, 2013. 27(1): p. 7-9.  McElwee, N.E. and R.W. Dubois, From methods to policy: Enthusiasm for rapid-learning health systems exceeds the current standards for conducting it. Journal of Comparative Effectiveness Research, 2013. 2(5): p. 425-7.  McIntosh, B., Developing a national learning health system. British Journal of Healthcare Management, 2017. 23(7): p. 304-305.  Moffatt-Bruce, S., et al., IDEA4PS: The Development of a Research-Oriented Learning Healthcare System. American Journal of Medical Quality, 2018. 33(4): p. 420-425.  Noritz, G., et al., "Learn From Every Patient": How a Learning Health System Can Improve Patient Care. Pediatric quality & safety, 2018. 3(5): p. e100.  Perla, R.J., B. Finke, and D.A. DeWalt, Learning Systems at Scale: Where Policy Meets Practice. JAMA, 2015. 314(20): p. 2131-2.  Psek, W.A., et al., Operationalizing the learning health care system in an integrated delivery system. EGEMS (Washington, DC), 2015. 3(1): p. 1122.  Roberts, D.W., Improving care and practice through learning health systems. Nursing Management, 2013. 44(4): p. 19-22.  Scoap Collaborative, W.G.f.t.S.C., et al., Creating a learning healthcare system in surgery: Washington State's Surgical Care and Outcomes Assessment Program (SCOAP) at 5 years. Surgery, 2012. 151(2): p. 146-52.  Serena, T.E., et al., A new approach to clinical research: Integrating clinical care, quality reporting, and research using a wound care network-based learning healthcare system. Wound Repair & Regeneration, 2017. 25(3): p. 354-365.  Shah, A., et al., Improving access to services through a collaborative learning system at East London NHS Foundation Trust. BMJ open quality, 2018. 7(3): p. e000337.  Sledge, G.W., et al., ASCO's Approach to a Learning Health Care System in Oncology. Journal of Oncology Practice, 2013. 9(3): p. 145-148.  Smoyer, W.E., P.J. Embi, and S. Moffatt-Bruce, Creating Local Learning Health Systems: Think Globally, Act Locally. JAMA: Journal of the American Medical Association, 2016. 316(23): p. 2481-2482.  Stucki, G. and J. Bickenbach, Functioning information in the learning health system. European journal of physical & rehabilitation medicine., 2017. 53(1): p. 139-143.  Sullivan, T., Improving quality and performance in Ontario's cancer services: lessons for constructing a learning healthcare system. Healthcare Quarterly, 2015. 17 Spec No(101208192): p. 5-9.  Wallace, P.J., et al., Optum Labs: Building A Novel Node In The Learning Health Care System. Health Affairs, 2014. 33(7): p. 1187-1194. |
| Global Level | Adams, S. and M.G. Titler, Building a learning collaborative. Worldviews on Evidence-Based Nursing, 2009. 7(3): p. 165-173.  Cresswell, K., S. Cunningham-Burley, and A. Sheikh, Creating a climate that catalyses healthcare innovation in the United Kingdom - learning lessons from international innovators. Journal of innovation in health informatics, 2017. 23(4): p. 882.  Holve, E. and C. Segal, Infrastructure to support learning health systems: are we there yet? Innovative solutions and lessons learned from American Recovery and Reinvestment Act CER investments. Journal of Comparative Effectiveness Research, 2014. 3(6): p. 635-45.  McCannon, C.J. and R.J. Perla, Learning networks for sustainable, large-scale improvement. Joint Commission Journal on Quality & Patient Safety, 2009. 35(5): p. 286-91.  McLinden, D., et al., The Learning Exchange, a Community Knowledge Commons for Learning Networks: Qualitative Evaluation to Test Acceptability, Feasibility, and Utility. JMIR formative research, 2019. 3(1): p. e9858.  Weaver, S.J., et al., A Collaborative Learning Network Approach to Improvement: The CUSP Learning Network. Joint Commission Journal on Quality & Patient Safety, 2015. 41(4): p. 147-59. |
