# Supplementary material for: Aiming for quality: a global compass for national learning systems
Source: Health Res Policy Syst. 2021 Jul 19;19:102. doi: 10.1186/s12961-021-00746-6 (PMC8287697; doi:10.1186/s12961-021-00746-6)
Supplement: Supplementary file 2 — Additional file 2. References of articles excluded during full-text review. [file 12961_2021_746_MOESM2_ESM.docx]

**Additional file 2: References of Articles Excluded During Full-Text Review**

Bindman, A.B., A Shared Responsibility for Developing a Learning Health System. Journal of Nursing Care Quality, 2017. 32(2): p. 95-98.

Deeny, S.R. and A. Steventon, Making sense of the shadows: priorities for creating a learning healthcare system based on routinely collected data. BMJ Quality & Safety, 2015. 24(8): p. 505-15.

Gaveikaite, V., et al., Learning Healthcare Systems: Scaling-Up Integrated Care Programs. Studies in Health Technology & Informatics, 2018. 247(ck1, 9214582): p. 825-829.

Koksma, J.-J. and J.A.M. Kremer, Beyond the Quality Illusion: The Learning Era. Academic medicine : journal of the Association of American Medical Colleges, 2019. 94(2): p. 166-169.

Kruk, M.E., et al., High-quality health systems in the Sustainable Development Goals era: time for a revolution. The Lancet Global Health, 2018. 6(11): p. e1196-e1252.

Mbuthia, D., et al., Kenyan health stakeholder views on individual consent, general notification and governance processes for the re-use of hospital inpatient data to support learning on healthcare systems. BMC medical ethics, 2019. 20(1): p. 3.

Munoz-Plaza, C.E., et al., Integrating qualitative research methods into care improvement efforts within a learning health system: addressing antibiotic overuse. Health Research Policy & Systems, 2016. 14(1): p. 63.

Nahm, E.-S., Mental Health Nurses: Are We Ready for a "Learning Health System"? Journal of the American Psychiatric Nurses Association, 2015. 21(4): p. 284-6.

Ovretveit, J., E. Nelson, and B. James, Building a learning health system using clinical registers: a non-technical introduction. Journal of Health Organization & Management, 2016. 30(7): p. 1105-1118.

Rees, P., et al., Quality improvement informed by a reporting and learning system. Archives of Disease in Childhood, 2014. 99(7): p. 702-3.

Rubin, J.C. and C.P. Friedman, Weaving together a healthcare improvement tapestry. Learning health system brings together health data stakeholders to share knowledge and improve health. Journal of Ahima, 2014. 85(5): p. 38-43.

Stoto, M., G. Parry, and L. Savitz, Analytical Methods for a Learning Health System: 4. Delivery System Science. EGEMS (Washington, DC), 2017. 5(1): p. 31.

Young, P.J., Learning Healthcare Systems Will Protect Patients from Unscientific Practice Variation. Annals of the American Thoracic Society, 2018. 15(2): p. 131-133.
