# Supplementary material for: Aiming for quality: a global compass for national learning systems
Source: Health Res Policy Syst. 2021 Jul 19;19:102. doi: 10.1186/s12961-021-00746-6 (PMC8287697; doi:10.1186/s12961-021-00746-6)
Supplement: Supplementary file 3 — Additional file 3. Summary of studies included by “Learning Level”. [file 12961_2021_746_MOESM3_ESM.docx]

**Additional file 3: Summary of Studies Included for the “Learning Level” Synthesis**

| Learning Level | Definition | Articles | Settings | Topics | Countries |
| --- | --- | --- | --- | --- | --- |
| Health Professional Level 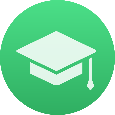 [This Photo](https://commons.wikimedia.org/wiki/File:Antu_applications-education-university.svg) by Unknown Author is licensed under [CC BY-SA](https://creativecommons.org/licenses/by-sa/3.0/) | Health students learning to develop a “health system view” of improvement. | 19 | Undergraduate Students (32%), Graduate Students (47%), and Trainees in Primary Care (11%), Psychiatry (5%), and Surgical Care (5%). | Topics included interprofessional learning, Practice-Based Learning (PBL), Systems-Based Practice (SBP), a combination of PBL and SBP, and learning systems. | Canada (5%), China (11%), Iran (5%), United States (74%), and United States-Sweden (5%). |
| Health Organizational Level 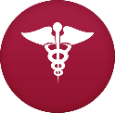 [This Photo](http://www.pngall.com/health-png) by Unknown Author is licensed under [CC BY-NC](https://creativecommons.org/licenses/by-nc/3.0/) | Health organizations developing the characteristics needed to form a learning system. | 15 | Acute Care (67%), Primary Care (7%), Acute and Primary Care (7%), Senior Care (7%) and General (13%). | Various characteristics of learning organizations including interprofessional learning, psychological safety, and knowledge sharing. | Ghana (7%), Ireland (7%), Norway (7%), Spain (7%), Sweden (7%), United Kingdom (7%), United States (40%) and Various (20%) |
| Sub-National/  National Level  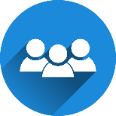 | Multiple health organizations learning together by participating in quality improvement initiatives. | 26 | Public Health (8%), Community Care (4%), Primary Care (27%), Acute Care (15%), Long-Term Care (4%), Integrated Care (15%), Specialized Services (15%) and Various (12%) | Topics ranged from adherence to specific clinical practice guidelines to broader improvements including integrated care. | Canada (4%), India (4%), Mozambique (4%), Rwanda (4%), United Kingdom (4%), United States (73%), United States-Canada (4%) and Various (4%) |
| Multiple Levels  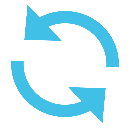 | Multiple stakeholders learning together to continuously improve the quality of health services based on the frontline experience. | 35 | Organizational Level (14%), Sub-National Level (14%), National Level (40%), Global Level (3%) and Non-Specific (29%). | Various topics including veteran health services, cancer care, cardiovascular disease, cerebral palsy, surgical care, mental health, and palliative care. | Australia (3%), Canada (3%), China (3%), Kenya (3%), Switzerland (3%), United Kingdom (3%), United States (77%), and United Kingdom-South Asia (3%), and General LMIC (3%). |
| Global Level 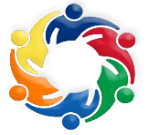 [This Photo](http://strenco.eu/) by Unknown Author is licensed under [CC BY-NC](https://creativecommons.org/licenses/by-nc/3.0/) | Multiple learning systems working together as a network. | 6 | Acute Care (67%) and Non-Specific (33%). | The common topic was having a platform to support peer-to-peer learning and scale knowledge. | United Kingdom (17%) and United States (83%). |
