# Supplementary material for: Aiming for quality: a global compass for national learning systems
Source: Health Res Policy Syst. 2021 Jul 19;19:102. doi: 10.1186/s12961-021-00746-6 (PMC8287697; doi:10.1186/s12961-021-00746-6)
Supplement: Supplementary file 4 — Additional file 4. Characteristics of studies included for the “Learning Systems” synthesis. [file 12961_2021_746_MOESM4_ESM.docx]

**Additional file 4: Characteristics of Studies Included for the “Learning Systems” Synthesis**

| # | Title | Country | Focus | Learning System Definition | Learning System  Features |
| --- | --- | --- | --- | --- | --- |
| 1 | A systems-based partnership learning model for strengthening primary healthcare. | Australia | National Level, Primary Care | A partnership learning model that uses an integrated quality improvement approach with an emphasis on systematically generating and using evidence for health system strengthening. | The Partnership Learning model included (1) comprehensive and integrated approach to healthcare improvement, (2) system-based research networks with multiple practices/services, managers, policy makers and others across the health system, (3) system-based participatory research with clinicians, office support staff, representatives of health-related organizations, managers, policy makers and community members, (4) translation of explicit knowledge (codified knowledge) and tacit knowledge (experience-based knowledge) where researchers collaborate with policy-makers and practitioners, (5) health systems strengthening where the ‘six building blocks’ of a health system are integrated (service delivery, health workforce, information, medical products/vaccines/technologies, financing, and leadership and governance), and (6) systems thinking because the health sector is a complex adaptive system (one that self-organizes, adapts and evolves with time). |
| 2 | Improving quality and performance in Ontario's cancer services: lessons for constructing a learning healthcare system. | Canada | Sub-National Level, Cancer Care | A platform for advancing knowledge in a specific area with strong clinical involvement, good planning and informatics capacity, strong political support and public reporting against goals. | This learning system for cancer care included (1) measures and incentives with a province-wide electronic platform to map the patient journey from prevention to palliative care, (2) accountability and contract agreements for cancer care, and (3) public reporting on performance measures across the continuum of cancer care where the public and policy-makers have access to the data they need for continuous learning and improvement. |
| 3 | Achieving best outcomes of patients with cardiovascular diseases in China by enhancing the quality of medical care and establishing a learning health care system. | China | National Level, Cardiovascular Disease | The capacity to monitor performance, learn about what works best for whom, and evaluate what strategies support successful implementation of best practices and achieve optimal outcomes. | This prototype for a national learning system includes (1) government leadership where some of the tertiary hospitals are designated as the leads in developing quality standards, collecting data, performing analyses, and sharing the results, (2) local evidence generation to learn from the front-line experience and inform the design of the healthcare system including policies and practices, (3) incentives for evidence application by monitoring compliance with clinical practice guidelines and creating policies that reward value, and (4) public reporting and transparency where the performance measures of each hospital are publicly available, with national/regional comparisons and benchmarks. |
| 4 | Approaching quality improvement at scale: a learning health system approach in Kenya. | Kenya | National Level, Acute Care | A mechanism for promoting continued improvement of basic hospital services, implementation of new effective practices and technologies, and conduct of locally relevant research to optimize interventions. | The Clinical Information Network consisted of (1) creating a network of stakeholders involved in the design, operation and governance, (2) using knowledge from routine clinical data for local improvements and wider health system performance monitoring, (3) promoting more rapid adoption of evidence into routine clinical care, and (4) supporting researchers to use the same data to conduct rapid and efficient health research that improves the delivery of health services. |
| 5 | Building Learning Health Systems to Accelerate Research and Improve Outcomes of Clinical Care in Low- and Middle-Income Countries. | LMIC | National Level, General | A system that is designed to generate and apply the best evidence for the collaborative health care choices of each patient and provider; to drive the process of discovery as a natural outgrowth of patient care; and to ensure innovation, quality, safety, and value in health care (adapted from the Institute of Medicine). | Building a learning system in a LMIC should cover (1) health system design that integrates the delivery of health services with the generation of new knowledge about the quality of these services, (2) research partnerships to promote research that matters for policy-makers and practitioners and helps strengthen trust, (3) stakeholder engagement that involves policy-makers, researchers, service providers, and patients in continuous learning and improvement, (4) information infrastructure where electronic health records include standardized data across multiple facilities, (5) data management and governance with opportunities to learn from international practices, and (6) global network on how to develop effective learning systems and share knowledge. |
| 6 | Functioning information in the learning health system. | Switzerland | National Level, Rehabilitation Services | A coordinated collection of functioning data across the three levels of health systems: the micro level of health services, the meso-level of health service programming, and the macro-level of policies that guide health service programming. | A nationwide learning system should cover (1) learning at the micro-level (individual rehabilitation plan) by having standardized documentation of a person’s functioning status, (2) learning at the meso-level (optimizing service provision) by ensuring that services are based on what matters to people and continuously monitoring performance along the continuum of care and across similar services, and (3) learning at the macro-level (rehabilitation policy and programming) to determine whether the rehabilitation system as a whole is producing the desired and expected impact at the population level. |
| 7 | Improving access to services through a collaborative learning system at East London NHS Foundation Trust. | United Kingdom | Sub-National Level, Mental Health and Community Health Services | A method to promote collaboration, accelerate learning, and provide improvement expertise to all teams, and senior sponsorship to help overcome barriers at all levels of the organization. | This learning system of community-based teams included (1) shared goals across all the teams, (2) measurement system, with standardized measures that were collected and shared transparently across all the teams, (3) methods to learn from each other, with face-to-face learning sets every 6 weeks, (4) support structure, with a project board, an executive sponsor for the whole learning system, local sponsors for each project and improvement advisors coaching each project team, and (5) shared theory of change, with a driver diagram created together to visualize how the teams believed they would improve access across their services.  This learning system resulted in reductions in waiting times from referral to first appointment (from an average of 60.6 days to 46.7 days, a 23% reduction), and non-attendance at first appointment (from an average of 31.7% to an average of 20.5%, a 36% reduction). |
| 8 | A learning health systems approach to improving the quality of care for patients in South Asia. | United Kingdom and South Asia | Global Level, Acute Care | An innovative approach to setting relevant priorities for improving the quality of care using routine clinical data captured through digital health information platforms. | The Care Quality Improvement Network consisted of (1) improving care by leveraging information captured through the electronic multi-centre acute and critical care surveillance platform, and (2) having structured discussions with frontline clinicians and stakeholders to select common quality improvement themes (process and outcome indicators, standardized assessment tools and treatment pathways). |
| 9 | ASCO's Approach to a Learning Health Care System in Oncology. | United States | General, Cancer Care | A system where science, informatics, incentives, and culture are aligned for continuous improvement and innovation—with best practices seamlessly embedded in the delivery process and new knowledge captured as an integral by-product of the delivery experience (adapted from the Institute of Medicine). | Learning systems should consider (1) capturing and understanding data from any source, (2) real-time clinical decision support, (3) measuring compliance in real time, (4) demonstrating that the data can be explored for the purpose of hypothesis generation and learning, and (5) de-identifying and entering the data in a manner compliant with applicable privacy legislation. |
| 10 | A new approach to clinical research: Integrating clinical care, quality reporting, and research using a wound care network-based learning healthcare system. | United States | Organizational Level, Wound Care | Knowledge is obtained continuously through routine clinical documentation at the point of care and turned into guidance through clinical decision support, with a resulting vast repository of data on treatment effectiveness to enhance RCTs and evidence-based medicine. | This for-profit learning system of wound care clinics (1) leveraged technology to standardize care based on real world evidence, (2) created electronic health records that collect all patient data at point-of-care and provide wound-specific clinical practice suggestions to standardize care, and (3) integrated research-related patient and wound data elements into the data collection framework of electronic health records. |
| 11 | Learn From Every Patient: implementation and early results of a learning health system. | United States | Organizational Level, Cerebral Palsy (Pediatric) | A system that is designed to generate and apply the best evidence for the collaborative health care choices of each patient and provider, to drive the process of discovery as a natural outgrowth of patient care, and to ensure innovation, quality, safety, and value in health care (adapted from the Institute of Medicine). | This local learning system integrated clinical care and research and used this new knowledge to continuously improve the quality of care which (1) drove evidence-based clinical quality improvement projects, facilitated research, and reduced healthcare costs, (2) resulted in a 27% reduction in hospitalizations, and (3) resulted in 176% to 210% reductions in healthcare costs compared to two control groups. |
| 12 | "Learn From Every Patient": How a Learning Health System Can Improve Patient Care. | United States | Organizational Level, Cerebral Palsy (Pediatric) | A continuous cycle of systematic care improvement by coupling evidence generation with evidence application to health care. | This local learning system supported the collection of research and clinical data together in electronic health records during routine clinical care to improve the practice of screening hip radiographs. This reduced the cost of care average ($66 per x-ray per child per year) and exposure to radiation (42–254 mRad per x-ray per year), |
| 13 | The Agency for Healthcare Research and Quality and the Development of a Learning Health Care System | United States | Organizational Level, General | Adopting evidence on a systematic basis and ensuring that it is incorporated into decision making throughout the organization in a consistent way. | Local learning systems should consider (1) directing support for health services research training toward individuals who work within health systems and in collaboration with the organization’s clinical and informatics leaders, (2) research health information technology on supporting the development of data analytic tools that can be used for population health management, and (3) supporting efforts to implement clinical decision support tools that can help practitioners recognize when their patients might benefit from evidence adopted across the organization. |
| 14 | Developing a framework for integrating health equity into the learning health system | United States | General | An integrated health system in which progress in science, informatics, and care culture align to generate new knowledge as an ongoing, natural by‐product of the care experience (adapted from the Institute of Medicine). | This learning system framework covers (1) person‐focused care, (2) privacy, (3) inclusiveness, (4) transparency, (5) accessibility, (6) adaptability, (7) governance, (8) cooperative and participatory leadership, (9) scientific integrity, and (10) value. |
| 15 | IOM Report: 'The Path to Continuously Learning Healthcare in America' | United States | General | Links personal and population data to researchers and practitioners, dramatically enhancing the knowledge base on effectiveness of interventions and providing real-time guidance for superior care in treating and preventing illness. | Learning systems can provide the best care at the lowest cost through (1) digital infrastructure to improve the capacity to capture clinical care and financial data, (2) data utility to improve research regulations, (3) clinical decision support, (4) patient-centered care, (5) community-clinical partnerships and services, (6) care continuity with and across organizations, (7) optimized operations, (8) financial incentives to reward continuous learning and improvement in the provision of best care at lower cost, (9) performance transparency, and (10) leadership support to continuously learn and improve the delivery of health services. |
| 16 | The emergence of a learning healthcare system. | United States | General | A system that is able to assess the risks and benefits of treatment options and learn from previous experiences and outcomes in determining beneficial options. | Learning systems should leverage data from electronic health records to (1) enhance the digital infrastructure, (2) enhance the utilization of data, and (3) enhance clinical decision support. |
| 17 | From methods to policy: Enthusiasm for rapid-learning health systems exceeds the current standards for conducting it. | United States | General | Quick learning using the growing data infrastructure to impact either clinical care or coverage/payment decisions. It follows an iterative process, conducted in real time and employs a rapid 'plan, do, check and act' cycle to address real-world knowledge gaps. | Rapid learning systems should (1) follow a set of consensus standards for rapid learning systems, (2) have the results undergo peer review before implementing rapid learning analyses into patient care decisions, (3) commit to reassessment of what happens after the 'change' has been implemented, and (4) make the findings publicly available to provide transparency and support other organizations considering similar healthcare improvements. |
| 18 | Developing a national learning health system. | United States | National Level, General | Data are simultaneously used – at macro level to inform policy and planning decisions, at meso level to improve the organization and delivery of care, and micro level to personalize care. | Learning systems should (1) have a national vision, (2) align major funders, (3) identify shared national goals, (4) establish baselines, and (5) evaluate the needs of stakeholders. |
| 19 | IDEA4PS: The Development of a Research-Oriented Learning Healthcare System. | United States | General | An organizational approach to health services delivery wherein efforts to improve both efficiency and effectiveness are grounded in experience with the populations served. | Learning systems should (1) build a shared language, (2) achieve stakeholder agreement to serve as champions, and (3) defer to the collective in exploring and expanding research and practice at the health system level. |
| 20 | Improving care and practice through learning health systems. | United States | General | Multiple stakeholders working interactively through a cloud of technology overlaid with a sense of shared governance and trust, a commitment to patient engagement, and a technologically supported means of iterative and rapid analysis of information and continuous dissemination of knowledge. | The process to develop a learning system includes (1) scanning and surveillance to assess the need for such a system, (2) inviting key stakeholders to participate in its development through an iterative design process, and (3) evaluating, adjusting, and disseminating the findings.  Learning systems should be (1) person-focused, (2) protect privacy, (3) include all interested parties in its governance, (4) be transparent and accessible, (5) adapt to changes rapidly, (6) be self-governing, cooperative, and participatory, (7) maintain a high level of scientific integrity, and (8) provide value to its participants. |
| 21 | Creating Local Learning Health Systems: Think Globally, Act Locally. | United States | Organizational Level,  General | Creating a continuous cycle of systematic care improvement by coupling evidence generation with evidence application. | Local learning systems could provide a model for developing learning systems at the regional, national, and international levels. They include (1) implementation of standardized care, (2) systematic collection of research data as part of all clinical visits, (3) opportunities for ongoing clinical and translational research publications, and (4) opportunities to participate in systematic improvement in the care of patients. |
| 22 | Optum Labs: Building A Novel Node In The Learning Health Care System. | United States | General | National and regional research networks that consist of multiple contributing nodes, or collaborations of providers and consumers of data or research. | The impact of learning systems depend on how well participants can align and integrate their efforts. This includes (1) drawing on the diverse perspectives and expertise of multiple health-related organizations, (2) providing access to the required data for the purposes of research, knowledge translation, and innovation, and (3) expanding the capacity for knowledge generation and providing a unique environment for testing new ideas to inform decisions about the policies and operational requirements. |
| 23 | PEDSnet: a National Pediatric Learning Health System. | United States | National Level, Inflammatory Bowel Disease (Pediatric) | A healthcare organization that is purposefully designed to produce research in routine care settings and implement evidence at the point of care. | The prototype for a national learning system includes (1) model for engaging participants called actor-oriented collaboration, (2) research, improvement, management, and patient care are intentionally integrated and “learning while doing” is the default, (3) active collaboration of all members of the system (from patients to clinicians to health system leaders), and (4) collaboration is supported by providing resources (e.g. web-based collaboration spaces, project management, and learning activities). |
| 24 | Rapid Learning: A Breakthrough Agenda. | United States | National Level, General | A system that generates useful knowledge to improve clinical care and translates new evidence into better health for millions of people. | Rapid learning systems support (1) building learning networks, delivery systems, and professional societies that use electronic health records and computerized databases to assess performance and adopt best practices, (2) research registries, databases, and learning networks for all diseases and subpopulations, (3) health care quality and efficiency, (4) personalized medicine, and (5) public health through a nationwide disease information system. |
| 25 | Fostering Collaboration Through Creation of an IBD Learning Health System. | United States | National Level, Inflammatory Bowel Disease | A cyclic framework designed to ensure the delivery of evidence-based care and the continuous improvement of that care and the outcomes it generates. | Learning systems should cover (1) patient-physician partnerships, (2) improvement efforts to create standardized approaches to care, (3) and supporting infrastructure including a model for improvement and various learning activities (e.g. monthly webinars, bi-annual in-person learning sessions, and a ListServ for email communications). |
| 26 | PEDSnet: How A Prototype Pediatric Learning Health System Is Being Expanded Into A National Network | United States | National Level, Inflammatory Bowel Disease (Pediatric) | A health care organization that is purposefully designed to produce research in routine care settings and implement evidence at the point of care. | Learning systems should support (1) learning as a community, (2) digital architecture to access data from electronic health records and patient registries, (3) quality improvement to improve care at the patient-level, (4) rapid research using registry data, (5) starting with a prototype that could be scaled into a national learning system, and (6) partnering with national data partners to create Big Data. |
| 27 | Learning Health Systems | United States | National Level, Pediatric | A system that merges healthcare delivery with research, data science, and quality improvement processes. It begins and ends with the clinician patient interaction, and aspires to provide continuous improvements in quality, outcomes, and healthcare efficiency. | Learning systems involve (1) active collaboration of all members of the system, from patients to clinicians to health system leaders, and success is defined by the impact of the system on the health and lives of patients. (2) having a learning cycle that begins at the point-of-care with the patient-clinician interaction, and (3) ongoing cycle between research and practice. |
| 28 | Ensuring public health's future in a national-scale learning health system. | United States | National Level, Public Health | A platform that seeks to leverage health data to allow evidence-based real-time analysis of data for a broad range of uses, including primary care decision making, public health activities, consumer education, and academic research. | National learning systems should cover (1) patient-centred care, (2) privacy, (3) inclusiveness, (4) transparency, (5) accessibility, (6) adaptability, (7) governance, (8) cooperative and participatory leadership, (9) scientific integrity, and (10) value. |
| 29 | Accelerating Research Impact in a Learning Health Care System VA’s Quality Enhancement Research Initiative in the Choice Act Era | United States | National Level, Veterans Health Services | A national network of implementation Programs, or “laboratories” to support efforts to implement research into practice more rapidly and consistently. | The national network of Evidence Synthesis Program Centres generates new evidence for implementation and builds capacity for routine data collection to inform national program implementation and evaluation. The focus is on implementation strategies to facilitate the uptake of best practices driven by evidence. The emphasis is on promoting access and improving quality, especially in lower resourced settings when the availability of effective treatments is uncertain. |
| 30 | Moving from discovery to system-wide change: the role of research in a learning health care system: experience from three decades of health systems research in the Veterans Health Administration. | United States | National Level, Veterans Health Services | A system where science, informatics, incentives, and culture are aligned for continuous improvement and innovation—with best practices seamlessly embedded in the delivery process and new knowledge captured as an integral by-product of the delivery experience (adapted from the Institute of Medicine). | Learning systems should cover (1) real-time access to knowledge, (2) digital capture of patient care experience, (3) engaged, empowered patients, (4) incentives aligned for value, (5) full transparency through public reporting, (6) leadership-instilled culture of learning, and (7) emphasis on system redesign and “lean” training. |
| 31 | A Person-Centered, Registry-Based Learning Health System for Palliative Care: A Path to Coproducing Better Outcomes, Experience, Value, and Science. | United States | General, Palliative Care | A system that generates and applies the best evidence for the collaborative health care choices of each patient and provider and drives the process of discovery as a natural outgrowth of patient care (adapted from the Institute of Medicine). | The process for developing a learning systems includes (1) assembling a codesign team to clarify aims, (2) learning from what others have already done, (3) tailoring the general model to the context, and (4) building a registry-based learning system using rapid cycle tests of change. |
| 32 | Operationalizing the learning health care system in an integrated delivery system. | United States | Sub-National Level, Acute Care | A system that utilizes sophisticated technologies and competencies to integrate clinical operations, research and patient participation in order to continuously generate knowledge, improve care, and deliver value. | Learning systems should focus on (1) data and analytics, (2) people and partnerships, (3) patient and family engagement, (4) ethics and oversight, (5) evaluation and methodology, (6) funding, (7) organization, (8) prioritization, and (9) deliverables. |
| 33 | Learning Systems at Scale: Where Policy Meets Practice. | United States | Sub-National Level, Primary Care | A systematic improvement framework to understand change across a mix of health systems and increase the likelihood of successful tests of change in payment policy and delivery of care. | Learning systems need to (1) establish clear aims, (2) develop an explicit theory of change, (3) create the context necessary for a test of the model, (4) develop the change strategy, (5) test the changes, (6) measure progress toward aim, and (7) plan for spread. |
| 34 | Creating a learning healthcare system in surgery: Washington State's Surgical Care and Outcomes Assessment Program (SCOAP) at 5 years. | United States | Sub-National Level, Surgical Care | A system that will improve care delivery by helping the experiences of other clinicians, patients, and informed stakeholders influence medical care received by any given individual. | The success of learning systems relies on (1) creating a sense of community between clinicians and hospitals that may not naturally exist, (2) creating financial incentives for volunteered time and collaborative work, and (3) accessing technology with integrated and functional electronic health records. |
| 35 | Using a network organizational architecture to support the development of Learning Healthcare Systems | United States | General | Clinical care, science, informatics, incentives and culture are aligned for continuous improvement, innovation and research; new knowledge is captured as a by-product of care, and evidence is applied reliably and is seamlessly embedded in the delivery process (adapted from the Institute of Medicine). | Learning systems should include (1) improving the outcomes that matter most to patients, families and clinicians which results in shared purpose, (2) data transparency regarding performance, (3) quality improvement methods, (4) infrastructure that creates connectivity across participants, and (5) collaborative infrastructure and standardized policies. |

*Notes: The content in this table includes direct quotations and/or paraphrases from the original source.*
